# Valorization of plant by-products in the biosynthesis of silver nanoparticles with antimicrobial and catalytic properties

**DOI:** 10.1007/s11356-024-32180-w

**Published:** 2024-01-26

**Authors:** Verónica Rocha, Pedro Ferreira-Santos, Cristina Aguiar, Isabel C. Neves, Teresa Tavares

**Affiliations:** 1 CEB - Centre of Biological Engineering, University of Minho, Campus de Gualtar, 4710-057 Braga, Portugal; 2 Department of Chemical Engineering, Faculty of Science, University of Vigo, As Lagoas, 32004 Ourense, Spain; 3 CBMA—Centre of Molecular and Environmental Biology, University of Minho, 4710-057 Braga, Portugal; 4 CQ-UM – Centre of Chemistry, University of Minho, Campus de Gualtar, 4710-057 Braga, Portugal; 5 LABBELS –Associate Laboratory, 4710-057 Braga/Guimarães, Portugal

**Keywords:** Plant by-products, Sustainability, Silver nanoparticles, Disinfection, Photodegradation, Cost analysis

## Abstract

Biosynthesis based on natural compounds has emerged as a sustainable approach for the production of metallic nanoparticles (MNP). The main objective of this study was to biosynthesize stable and multifunctional silver nanoparticles (AgNP) using different plant by-products as reducers and capping agents. Extracts obtained from *Eucalyptus globulus, Pinus pinaster, Citrus sinensis*, *Cedrus atlantica* and *Camellia sinensis* by-products, were evaluated. From all plant by-products tested, aqueous extract of eucalyptus leaves (EL), green tea (GT) and black tea (BT) were selected due to their higher antioxidant phenolic content and were individually employed as reducers and capping agents to biosynthesize AgNP. The green AgNP showed zeta potential values of -31.8 to -36.3 mV, with a wide range of particle sizes (40.6 to 86.4 nm), depending on the plant extract used. Green AgNP exhibited an inhibitory effect against various pathogenic bacteria, including Gram-negative (*P. putida, E. coli*, *Vibrio spp.*) and Gram-positive (*B. megaterium, S. aureus, S. equisimilis*) bacteria with EL-AgNP being the nanostructure with the greatest antimicrobial action. EL-AgNP showed an excellent photodegradation of indigo carmine (IC) dye under direct sunlight, with a removal percentage of up to 100% after 75 min. A complete cost analysis revealed a competitive total cost range of 8.0–9.0 €/g for the biosynthesis of AgNP.

**Graphical Abstract:**

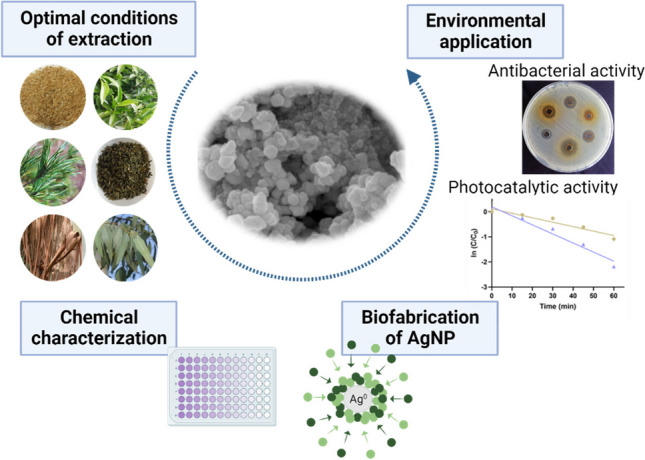

**Supplementary Information:**

The online version contains supplementary material available at 10.1007/s11356-024-32180-w.

## Introduction

Metallic nanoparticles (MNP) are an unusual class of materials with distinctive properties and wide applications in numerous fields (Khan et al. 2019). Surface Plasmon Resonance (SPR), a high surface-to-volume ratio, size, reconfigurable geometry, surface charge, wide band gap, generation of reactive oxygen species (ROS) and intrinsic stability are some of the characteristics of these MNP (Yaraki et al. 2022). One of the most interesting examples of these nano-entities are silver nanoparticles (AgNP) due to their excellent conductivity, stability and multiple applications as catalysts, antiviral, antifungal and antibacterial agents (Yaqoob et al. 2020; Esmaili et al. 2020; Salem and Fouda 2021).

One of the most pressing demands in the field of environmental remediation is the development of new and green methodologies for MNP synthesis, taking into consideration the aspects of environmental sustainability, innovative, simple and economical products (Rani et al. 2020; Bashir et al. 2023; Rocha et al. 2024; Chen et al. 2024). Various bioresources such as plants, algae, bacteria, yeasts and fungi either as reducing and as capping agents, are utilized in green synthesis of MNP (Singh et al. 2018; Roychoudhury 2020; Annamalai et al. 2021; Li et al. 2021; Adebayo et al. 2021). Within the green methods, plant biodiversity allowed various new syntheses by using plant extracts to biosynthesize MNP due to a rapid and simple technique and scalability of production (Pyrzynska and Sentkowska 2022; Nguyen et al. 2022; Ijaz et al. 2022).

Plant extracts including those from eucalyptus leaves (Rocha et al. 2023), eucalyptus bark (Das 2021), orange peels (Skiba and Vorobyova 2019), black plums (Parmar et al. 2019), kidney beans (Rani et al. 2020), black tea (Göl et al. 2020) and green tea (Kharabi Masooleh et al. 2019) among others have been successfully used to biosynthesize AgNP. There are some advantages of using plant extracts, namely the capping ability induced on the MNP by the polyphenol extract matrix, extending their reactivity and the decreased toxicity of the reducing agent compared to the toxicity of the chemicals utilized in other procedures. Additionally, due to the extracts' high water solubility, they can serve as a source of nutrients to promote complementary biodegradation and the natural product valorization, which in some situations are regarded as wastes with no additional value (Kuppusamy et al. 2016; Balamurugan and Saravanan 2017; Radwan et al. 2020; Mehwish et al. 2021).

Water treatment is a global issue and most conventional approaches have several drawbacks. There is an urgent need for the development of sustainable, efficient and cost-effective materials/methods for wastewater treatment. Extract-mediated AgNP have been used as an agent for effective environmental remediation, including the removal/degradation of biological and non-biological contaminants. Rani et al. 2020 have shown the excellent multifunctions (photocatalytic, catalytic and antimicrobial) of AgNP prepared using kidney beans (*Phaseolus vulgaris*) extract as reducing and stabilizer agent. Also, Mehwish et al. 2021 showed the efficient sunlight photodegradation of organic dyes and the excellent antimicrobial action against pathogenic bacteria, presented by AgNP biosynthesized using *Moringa oleifera* seed extract.

To date, only a few studies have reported comparative evaluations of the effect of different plant extracts on the synthesis of AgNP (Paosen et al. 2017; Salayová et al. 2021; Balčiūnaitienė et al. 2022). In the present study, a screening involving a range of different aqueous plant extracts obtained from *Eucalyptus globulus, Pinus pinaster, Citrus sinensis*, *Cedrus atlantica* and *Camellia sinensis* was performed to select the best extracts to synthesize stable and multifunctional AgNP. Optimal conditions of extraction (raw material, extraction time and temperature) were determined using as a basis the total phenolic and flavonoid content of each extract. The bioinspired synthesized AgNP were evaluated as antibacterial agents against various pathogenic bacteria and as alternative photocatalysts for the degradation of indigo carmine (IC) dye in an aqueous solution under sunlight. For the first time, a complete cost analysis for the biosynthesis of AgNP was carried out to evaluate its cost-effectiveness and scalability of production.

## Materials and methodologies

A detailed description of the materials and the methodologies used in this work are described in Supplementary Material.

### Chemical characterization of plant extracts

#### Determination of total phenolic content (TPC) and total flavonoid content (TFC)

The TPC and TFC were used as selection criteria to choose the most promising extracts to synthesize highly stable AgNP.

The TPC of the aqueous extracts was accessed using the Folin-Ciocalteu colorimetric assay at 700 nm (Singleton and Rossi 1965). Using a standard curve of gallic acid (10–500 mg/L, R^2^ = 0.9990), the TPC was calculated as mg of gallic acid equivalents (GAE) per gram of dry plant material (mg_GAE_/g).

The aluminium chloride (AlCl_3_) colorimetric assay was used to quantify the TFC of extracts at 510 nm (Ferreira-Santos et al. 2020). Using a standard curve of catechin (10–600 mg/L, R^2^ = 0.9990), the TFC was calculated as mg of catechin equivalents (CE) per gram of dry plant material (mg_CE_/g).

#### Determination of antioxidant activity

To examine the antioxidant capacity of the extracts, three independent methods were used: the ferric reducing antioxidant power (FRAP assay), the free radical scavenging activity (DPPH assay) and the radical cation decolorization (ABTS assay).

The FRAP assay was performed using the Benzie and Strain methodology (Benzie and Strain 1999). Absorbance was measured at 593 nm using water as a blank. Based on a standard curve of FeSO_4_.7H_2_O (100–1250 µm/L, R^2^ = 0.9990), the ability of the extracts to reduce Fe(III) was calculated as millimoles of ferrous equivalent per gram of dry plant material (mmol_Fe(II)_/g).

The DPPH free radical scavenging activity was assessed using the method described by Ferreira-Santos et al. (Ferreira-Santos et al. 2019). Absorbance was measured at 515 nm using water as a blank. Trolox (6-hydroxy-2,5,7,8-tetramethylchroman-2-carboxylic acid) standard solution was used to perform a calibration curve (10–400 µM, R^2^ = 0.9980). The DPPH values were recorded as millimoles of Trolox equivalent (TE) per gram of dry plant material (mmol_TE_/g).

The ABTS radical scavenging activity of extracts was assessed according to Ferreira-Santos et al*.* (Ferreira-Santos et al. 2019). Water was used as a blank to test the absorbance at 734 nm. A standard solution of Trolox was used to build the calibration curve (30–800 µM, R^2^ = 0.9960) and results were expressed as millimoles of Trolox equivalent (TE) per gram of dry plant material (mmol_TE_/g).

### Biofabrication of AgNP

For the green synthesis of AgNP, 100 mL of selected extracts of eucalyptus leaves, green tea and black tea (10% w/v) were individually added dropwise to 100 mL of 60 mM AgNO_3_ (with constant stirring at 50 °C for 1 h). Therefore, the mass of silver used was 0.6350 g which corresponds to 5.89 initial millimoles of silver. The reaction mixture gradually darkened in color, indicating the production of AgNP. Finally, the obtained suspensions of AgNP were separated by centrifugation at 10,000 rpm for 30 min to remove the unreacted Ag^+^ ions and extract residues. The precipitate was washed three times with ultrapure water and absolute ethanol and finally lyophilized. The biosynthesized AgNP were characterized. More information is found in the Supplementary material.

### Antibacterial activity

The antibacterial activity of selected extracts and biosynthesized AgNP was investigated against both Gram-negative bacteria, *Escherichia coli* (ATCC 25922), *Pseudomonas putida* S12 (ATCC 700801) and *Vibrio spp*. (CECT 7119) and Gram-positive bacteria, *Bacillus megaterium* (ATCC 14581) *Staphylococcus aureus* (ATCC 6538) and *Streptococcus equisimillis* (CECT 926). Nutrient broth medium was used for the cultivation of freshly growing bacteria (turbidity of 0.5 McFarland suspension). Using sterilized cotton swabs, 100 µL of each cell suspension was spread on a nutrient agar surface. The agar medium was then punched with 6 mm diameter wells and filled with 50 µL of biosynthesized AgNP aqueous solutions (1 mg/mL) as well as with the respective aqueous extracts. Subsequently, the plates were maintained at 37 °C for 24 h and the antibacterial action was determined by measuring the diameter of the growth inhibition zone (or halo), in mm. Each test was performed in triplicate.

### Photocatalytic activity

The photocatalytic capacity of green AgNP to degrade indigo carmine (IC) dye was evaluated under sunlight. An aqueous solution of IC (10 mg/L) with 0.5 or 1 g/L of AgNP was first placed in the dark for 30 min under magnetic stirring, to allow the equilibrium to be reached before starting the photocatalytic reaction. Thereafter, the solution was exposed to sunlight from 1 to 3 h *p.m*. Centrifugation was used to separate the AgNP from the dye solution after exposure and a UV–Vis spectrophotometer was used to examine the supernatant at 610 nm. Control experiments with and without catalyst in dark and sunlight conditions were also executed to determine the effects of catalyst and sunlight per se. Each experiment was done in triplicate.

The IC dye degradation efficiency was determined as follows:

1 $$\mathrm{Degradation\, efficiency\, }\left(\mathrm{\%}\right)= \frac{{{\mathrm{C}}}_{0}-{{\mathrm{C}}}_{{\text{t}}}}{{{\mathrm{C}}}_{{\mathrm{o}}}}\times 100$$

where C_0_ is the initial solution concentration (mg/L) and C_t_ is the final solution concentration at time t (mg/L).

## Results and discussion

### Selection and characterization of aqueous plant extracts

Aqueous extraction from different plant by-products, like eucalyptus bark and leaves, pine needles, orange leaves, cedar wood, as well as commercial green and black tea, was carried out to assess the potential of resulting extracts for biosynthesizing AgNP. From the perspective of circular economy and green development, the use of natural materials for the synthesis of MNP recycled in the treatment of wastewater is the most appealing one, according to the twelve principles of Green Chemistry (Anastas and Eghbali 2010). The use of biowastes in chemical synthesis can help to clean up the environment while producing new functional materials (Dugmore et al. 2017).

The establishment of extraction conditions is important because an inadequate method for the recovery of bio-compounds can affect the content and the functional properties of obtained extracts to be involved in the synthesis of MNP (Soltys et al. 2021; Martínez-Cabanas et al. 2021). Water was selected as the extraction solvent as it is the safest, cheapest and most environmental friendly solvent, and its efficiency has previously been reported in terms of phenolic compounds and other antioxidants extraction, using conventional or modern techniques (Kuppusamy et al. 2016; Bilal et al. 2019; Salayová et al. 2021; Martínez-Cabanas et al. 2021).

**Fig. 1:**
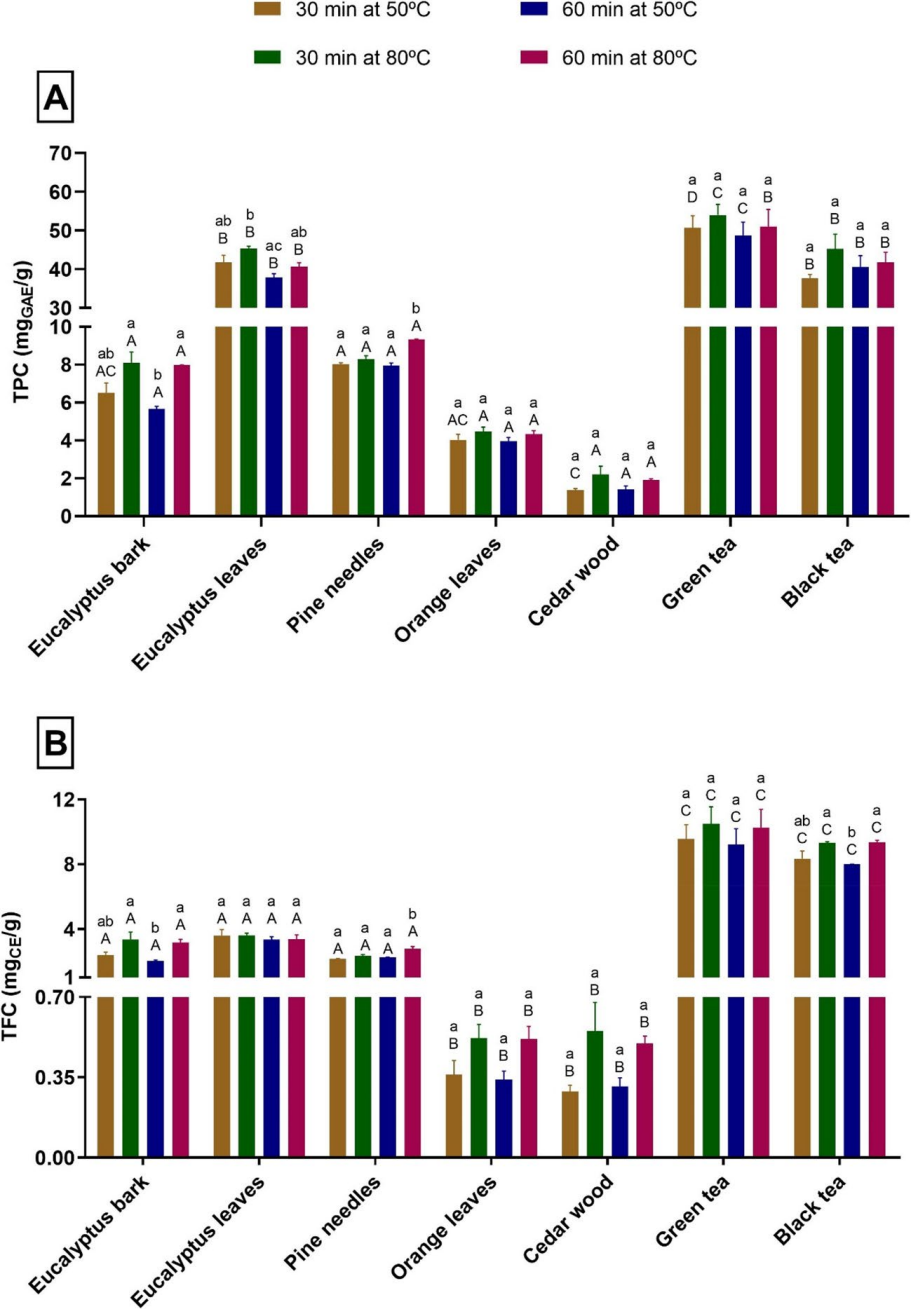
Total phenolic content (TPC) (**A**) and Total flavonoids content (TFC) (**B**) of plant extracts. Values are expressed as mean ± SD. Different lowercase letters show significant differences (*p* < 0.05) between extraction conditions for the same plant by-product. Different capital letters show significant differences (*p* < 0.05) between plant extracts for the same extraction conditions

For a clearer comparison between the obtained extracts, the total phenolic (TPC) and flavonoid (TFC) contents were determined for different extraction conditions. Figure 1 shows the results obtained for each aqueous extract at different temperatures (50 and 80 °C) and times (30 and 60 min) of extraction. The extracts obtained from eucalyptus leaves (40.7 to 45.3 mg_GAE_/g), green tea (48.7 to 54.0 mg_GAE_/g) and black tea (37.6 to 45.2 mg_GAE_/g) are the ones with the highest TPC and those from cedar wood (1.4 to 2.2 mg_GAE_/g) have the lowest TPC, regardless the extraction conditions.

As it may be observed in Fig. 1A, the TPC value of the extract obtained at 50 °C and 30 min is not statistically different (*p* > 0.05) from those of the extracts obtained within other conditions, except for pine needles extract obtained at 80 °C for 60 min, which presented a higher phenolic content (9.3 mg_GAE_/g). Several authors such as Machado et al*.* (Machado et al. 2013), optimized the extraction conditions (temperature, time) for leaves from 26 different tree species and concluded that the highest temperature (80 °C) presented the greatest outcomes for the extraction of phenolic compounds, without significant degradation of these antioxidants. For some types of plant leaves, extraction times of 20 min are sufficient to obtain almost all of the antioxidants, being influenced by the temperature used in the process. Moreover, Chandini et al*.* (Bilal et al. 2019) demonstrated that extraction times higher than 40 min at 90 °C can degrade some phenolic compounds (*e.g.* flavins and catechins) in the extracts of black tea.

The profile of TFC (Fig. 1B) is similar to the one of TPC and the highest values were reached for the green tea and the black tea extracts with maximum values of 10.5 and 9.3 mg_CE_/g, respectively. The eucalyptus leaves extract only reached a maximum value of 3.7 mg_CE_/g, a value similar to eucalyptus bark and pine needles extracts. This is a low value in comparison to the one described by Kuppusamy et al*.* (Kuppusamy et al. 2016), who obtained a TFC of 13.7 ± 0.9 mg_QE_/g (quercetin equivalents (QE) in mg per g of dry material) for eucalyptus leaves. On the other hand, Balčiūnaitienė et al*.* (Balčiūnaitienė et al. 2022) obtained a lower TFC of 0.48 ± 0.04 mg_RE_/g (rutin equivalents (RE) in mg per g of dry plant material) for dry *Eucalyptus globulus* leaves. These differences may be due to the extraction conditions (boiled water, sample/solvent ratio, time, etc.), as well as to the provenance and storage conditions of the raw material.

Therefore, based on the high TPC and TFC, the eucalyptus leaves, green tea and black tea extracts obtained at 50 °C for 30 min (extracts obtained with lower associated energy costs) were selected as promising candidates to synthesize AgNP. Similarly, Salgado et al. (2019) studied the effect of phenolics present in plant extracts on the synthesis of MNP and selected the extracts with higher phenolic content for the synthesis of iron oxide nanoparticles, including eucalyptus leaves extract.

There are many distinct compounds in plant extracts that function as antioxidants through different reaction mechanisms (Balčiūnaitienė et al. 2022). Therefore, it is strongly advised that at least two distinct methodologies be used to determine the antioxidant capacity of plant extracts (Prior et al. 2005). In this study, three different methods were employed to assess the antioxidant activity of the selected extracts from eucalyptus leaves, green tea and black tea (Table 1).

**Table 1:** Antioxidant activity of selected plant aqueous extracts

Plant Extract	Eucalyptus leaves	Green tea	Black tea
FRAP (mmol_Fe(II)_/g)	0.910 ± 0.023^AB^	1.188 ± 0.052^A^	0.770 ± 0.118^B^
ABTS (mmol_TE_/g)	1.909 ± 0.167^A^	3.099 ± 0.102^B^	2.034 ± 0.343^A^
DPPH (mmol_TE_/g)	2.550 ± 0.090^A^	3.080 ± 0.047^B^	2.310 ± 0.150^A^

Values are expressed as mean ± SD. Different capital letters show significant differences (*p* < 0.05) between plant extracts for the same antioxidant assay

The ability of the eucalyptus leaves, green tea and black tea extracts to reduce Fe3 + was evaluated using the FRAP method, and the results showed a reduction capacity of 0.910, 1.188 and 0.770 mmol Fe(II)/g, respectively. These data were higher than that reported for Eucalyptus globulus extract (Salgado et al. 2019). The free radical scavenging activity (DPPH and ABTS methods) are spectrophotometric methods employed for the assessment of the antioxidant activity of various compounds present in vegetables, beverages, foods, or extracts. The DPPH method is based on the ability of antioxidants to reduce the stable free radical (2,2-diphenyl-1-picrylhydrazyl, DPPH•) to the respective hydrazine, and the ABTS test is based on the capacity of antioxidants to inhibit the radical cation ABTS• + absorbance. These tests are reliable, accurate, fast, simple and cost-effective ways to assess the capacity of natural antioxidants to scavenge free radicals. Regarding the DPPH and ABTS results, the highest antioxidant activity was attained for the green tea extracts (about 3 mmolTE/g by both methods), compared to eucalyptus and black tea extracts (Table 1). Martínez-Cabanas et al. (2021) studied the antioxidant capacity of eleven plant extracts by the DPPH method and obtained a value above 5 mmolTE/L for eucalyptus (Eucalyptus globulus) and for green tea (Camellia sinensis), a lower activity compared to that obtained in the present work, 255.0 mmolTE/L (2.550 ± 0.090 mmolTE/g) for eucalyptus leaves and 308.0 mmolTE/L (3.080 ± 0.047 mmolTE/g) for green tea extracts. These results show the positive correlation of antioxidant activity assessed by the FRAP, DPPH and ABTS tests with the TPC and TFC of plant extracts, indicating the contribution of extracted biomolecules to the biological activity and it is in agreement with previous results (Salgado et al. 2019; Kędzierska-Matysek et al. 2021; Martínez-Cabanas et al. 2021).

### Green synthesis of AgNP

AgNP were successfully produced by the reduction of AgNO_3_ with the phenolic-rich aqueous extracts of eucalyptus leaves (EL-AgNP), green tea (GT-AgNP) and black tea (BT-AgNP) previously obtained (50 °C for 30 min) and chemically characterized (Fig. 1 and Table 1).

**Fig. 2:**
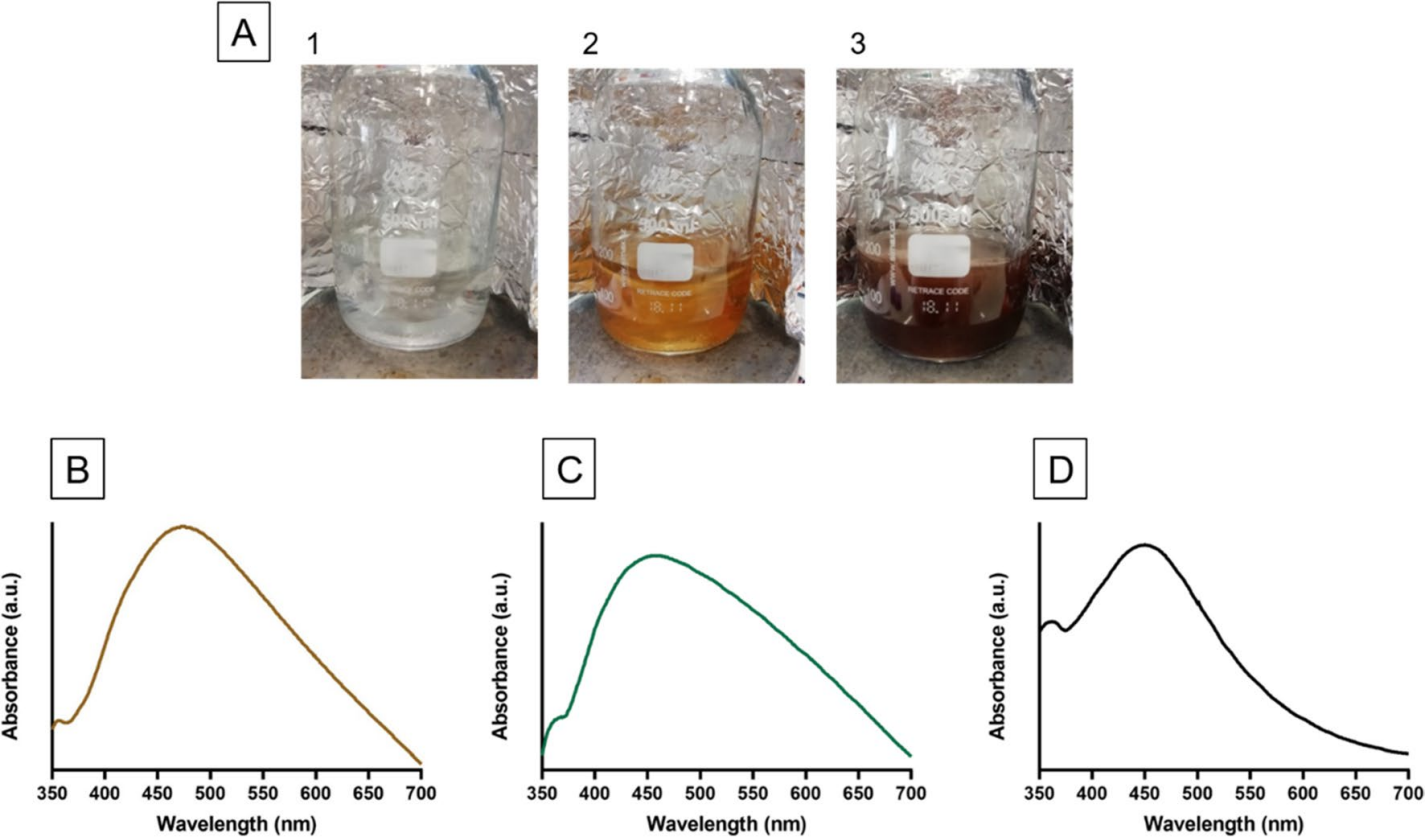
Changes in the color of the colloidal solutions indicate the formation of AgNP (**A**) 1. Silver nitrate solution, 2. Addition of eucalyptus leaves extract to silver nitrate solution at zero time (start of reaction) and 3. After 1 h of reaction at 50 °C (end of reaction). UV–visible spectra of AgNP synthesized by aqueous extracts from (**B**) eucalyptus leaves (EL-AgNP), (**C**) green tea (GT-AgNP) and (**D**) black tea (BT-AgNP)

The color change interpretation is regarded as the preliminary optical inference for the AgNP synthesis. The addition of phenolic extracts to AgNO_3_ solution at 50 °C (ratio of 1:1) transformed the color of the suspension from colorless to pale yellow, then to a dark brownish-red (Fig. 2A). The appearance of brownish is due to the reduction of the Ag^+^ ions to Ag^o^ by phenolic extract, evidenced by UV–visible spectroscopy (Fig. 2B) (Mo et al. 2015). SPR occurs when free electrons on the metal surface generate the surface plasmon as a consequence of beaming MNP at a specific refraction angle and the intensity of the reflected light decreases (Noguez 2007; Tayyab et al. 2022). The green AgNP exhibited SPR vibration bands at 474, 453 and 450 nm confirming the synthesis of EL-AgNP, GT-AgNP and BT-AgNP, respectively (Fig. 2B) and this is in agreement with previous results (Dugmore et al. 2017; Kharabi Masooleh et al. 2019).

**Fig. 3:**
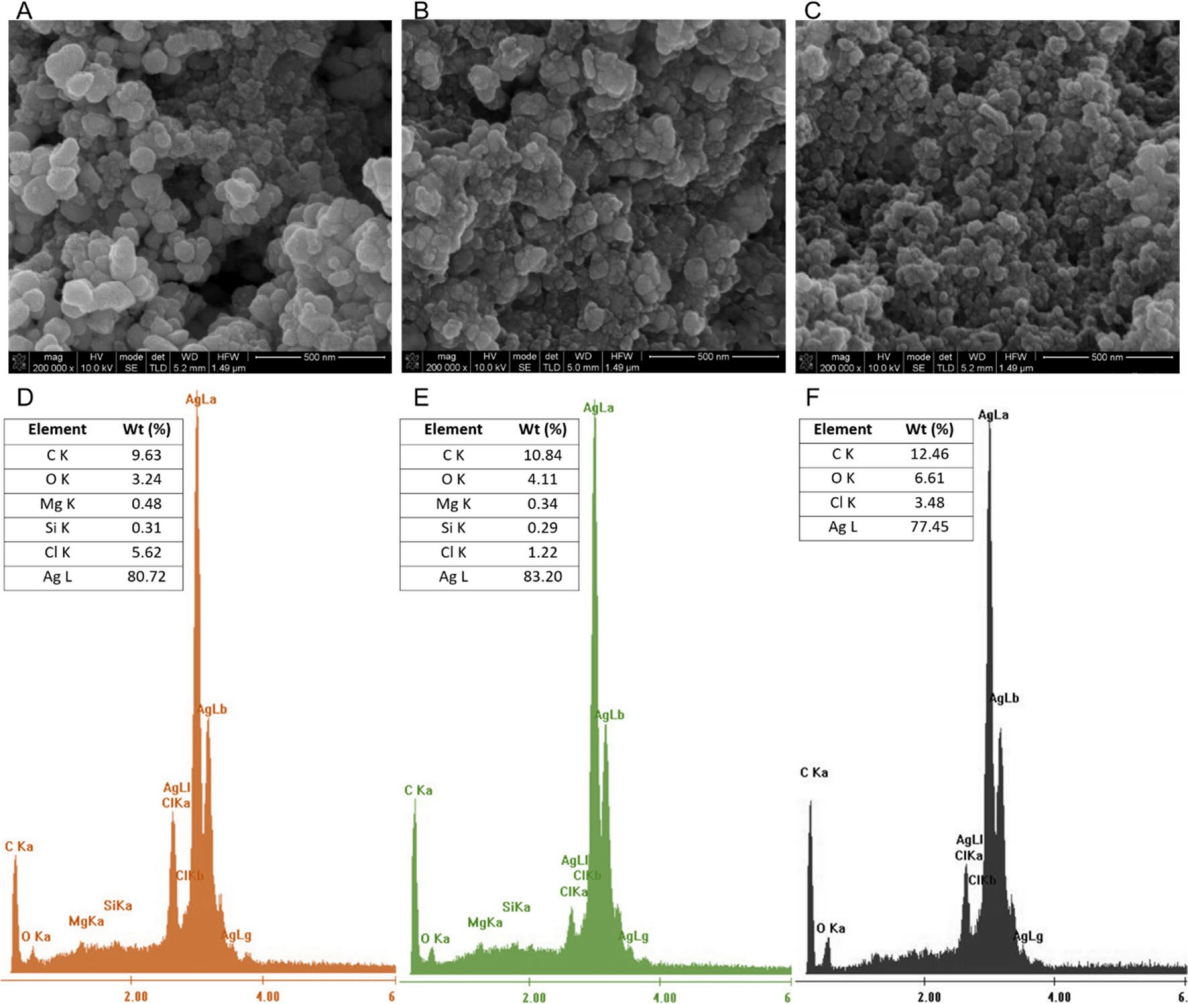
SEM images of AgNP synthesized by eucalyptus leaves (EL), green tea (GT) and black tea (BT) extracts: (**A**) EL-AgNP, (**B**) GT-AgNP and (**C**) BT-AgNP. EDX spectra of (**D**) EL-AgNP, (**E**) GT-AgNP and (**F**) BT-AgNP

SEM was performed to investigate the surface morphology of the biosynthesized AgNP with different aqueous extracts. AgNP morphology is homogeneous with spherical particles (Fig. 3A-B). The size distribution is in the range of 29–136, 14–81 and 22–81 nm for the synthesized EL-AgNP, GT-AgNP and BT-AgNP, respectively. The calculated average particle-size distribution (average of 25 particles) suggests that the BT-AgNP were the smallest ones (40.6 ± 16.0 nm), followed by the GT-AgNP (47.1 ± 21.1 nm) and, finally, by the largest EL-AgNP (86.4 ± 28.2 nm). Devatha et al*.* (Devatha et al. 2016) proposed that the amounts and types of phenolic compounds are two of the main reasons for the variability in the sizes of MNP. It was suggested that a high concentration of reductive biomolecules present in plant extracts leads to rapid production of AgNP and their subsequent growth via Ostwald reopening, which leads to an increase in the size of the AgNP over time (Rani et al. 2020). In this work, green tea extract showed the highest TPC (50.7 ± 3.1 mg_GAE_/g) and TFC (9.6 ± 0.9 mg_CE_/g), but EL-AgNP presented the highest size (86.4 ± 28.2 nm), indicating the variability of the phenolic compounds in each extract.

The EDX spectra of EL-AgNP, GT-AgNP and BT-AgNP are shown in Fig. 3D-E. The presence of silver at 3 keV was detected, confirming the effective formation of AgNP, due to SPR metallic AgNP (Bilal et al. 2019). Other authors described that the formation of spherical-shaped AgNP provides a peak in the range of 2.5–3.5 keV (Taruna et al. 2016), corroborating our results. Also, the presence of magnesium, oxygen and chloride resulting from the organic compounds present in extracts was identified, being responsible for Ag ions reduction and stabilization of resultant AgNP (Oluwaniyi et al. 2016; Alghoraibi et al. 2020). Accordingly, wt (%) values determined in EDX analyses revealed that GT-AgNP retained 83.2% of silver (4.90 mmol of Ag), EL-AgNP retained 80.7% (4.75 mmol) and BT-AgNP retained 77.5% of silver (4.56 mmol). These results are in concordance with the obtained by the FRAP method, indicating that the green tea extract shows stronger reducing activity, followed by eucalyptus leaves and black tea.

The number of atoms per nanoparticle (N) was calculated using a procedure described by Kalishwaralal et al*.* (Kalishwaralal et al. 2010) and Rani et al. (2020), Eq. 2:

2 $$N=\frac{\pi \rho {D}^{3}}{6M}{N}_{A}$$

where ρ is the density of the face-centered cubic crystalline (fcc) structure of silver (10.5 g/cm^3^), D is the average diameter of EL-AgNP (8.6 $x$ 10^–6^ cm), GT-AgNP (4.7 $x$ 10^–6^ cm) and BT-AgNP (4.1 $x$ 10^–6^ cm), M is the molar mass of silver (107.87 g) and N_A_ is the Avogadro’s number, 6.023 × 10^23^, assuming 100% conversion of all silver ions to AgNP. The N calculated was 19.5 × 10^6^ (EL-AgNP), 3.19 × 10^6^ (GT-AgNP) and 2.11 × 10^6^ (BT-AgNP), suggesting that EL-AgNP have more silver atoms per nanoparticle with a high average diameter.

The molar concentration of AgNP (C) was calculated using Eq. 3 (Kalishwaralal et al. 2010; Rani et al. 2020):

3 $$C=\frac{{N}_{T}}{NV{N}_{A}}$$

where N_T_ is the total number of silver atoms (number of mol of silver ions quantified by EDX × Avogadro’s number), N is the average number of atoms per nanoparticle (Eq. 2) and V is the total volume of solution used in the biosynthesis (0.2 L). Therefore, the molar concentration was 1.22 nM (EL-AgNP), 7.68 nM (GT-AgNP) and 10.8 nM (BT-AgNP). These values show that the AgNP from the EL extract has a lower concentration than the other particles prepared with the leaves from the different teas.

Zeta potential is the physical property that measures the effective electric charge on the MNP surface and it is a crucial parameter for the stability characterization of AgNP in aqueous suspensions. The repulsive interaction between the particles is determined by the surface charge as the measured zeta potential values indicate the particles' tendency to agglomerate or to disperse (Ruíz-Baltazar et al. 2018). The synthesized AgNP showed high zeta potential values of -31.8, -35.3 and -36.3 mV for El-AgNP, GT-AgNP and BT-AgNP, respectively compared to results obtained by other authors (Paosen et al. 2017; Kharabi Masooleh et al. 2019). The high negative values indicate the coordination of anionic stabilizing agents and the stability of the AgNP colloidal solution as a result of electrostatic repulsion between the negative charges (Paosen et al. 2017).

**Fig. 4:**
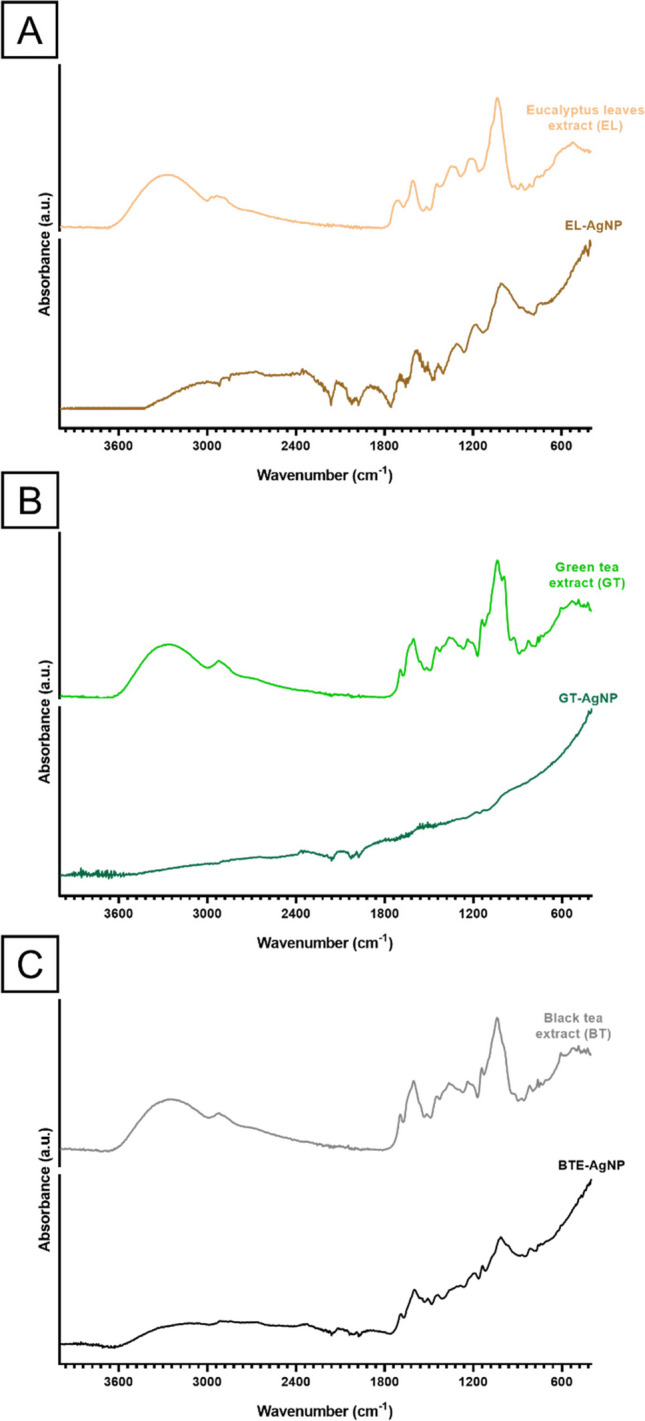
FTIR of extracts from eucalyptus leaves (EL) (**A**), green tea (GT) (**B**) and black tea (BT) (**C**) and biosynthesized AgNP: EL-AgNP (**A**), GT-AgNP (**B**) and BT-AgNP (**C**)

The FTIR spectra of aqueous extracts and the respective biosynthesized AgNP are shown in Fig. 4.

In the aqueous extracts, the bands between 3600 and 3000 cm^−1^ represent the O–H stretching vibration of the hydroxyl group (− OH) in polysaccharides and phenolic compounds and are assigned to secondary amides (− NH) (Ruíz-Baltazar et al. 2018). The band situated at 2923 cm^−1^ is associated with the C − H characteristic stretching vibration from CH and CH_2_ present in the aliphatic compounds (Perugu et al. 2016). The band at 1634 cm^−1^ represents the carbonyl group C═O stretch vibration of aromatic ketones, amine and carboxyl which are typically found in the proteins from plant extract (Bergal et al. 2022). The N–H functional groups of primary and secondary amides are responsible for the absorption band at 1515 cm^−1^. The peak at 1492 cm^−1^ can be related to the C═C stretching of the aromatic ring of the lignin (Shanmugam et al. 2016). The intensity band located at 1422 cm^−1^ is related to the symmetric bending of the CH_2_ which is present in comparable compounds such as cellulose, while the bands located at 1379 and 1320 cm^−1^ correspond to the bending vibration of C-O and C-H groups of the aromatic ring of the polysaccharides (de Jesús Ruíz-Baltazar et al. 2017). A band situated at 1062 cm^−1^ is attributed to the C-N stretching vibration band of aliphatic amines and the presence of a C-O stretching vibration was shown by the band at 1043 cm^−1^. This band could be used to assign a primary, secondary or tertiary structure of alcohol, that establishes the existence of phenolic compounds or ether and hydroxyl groups in cellulose (Manikandan et al. 2015; Pham et al. 2020). The bands at 922, 872, 765 and 608 cm^−1^ were associated to out of plane C-H bending vibrations in aromatics and alkenes (Esmaili et al. 2020). All the identified bands are common in the spectra of the biosynthesized AgNP, especially in the case of EL-AgNP and BT-AgNP. These results support that these functional groups play essential roles in the reduction of Ag^+^ ions to biosynthesize AgNP which is consistent with other works (Pham et al. 2020).

**Fig. 5:**
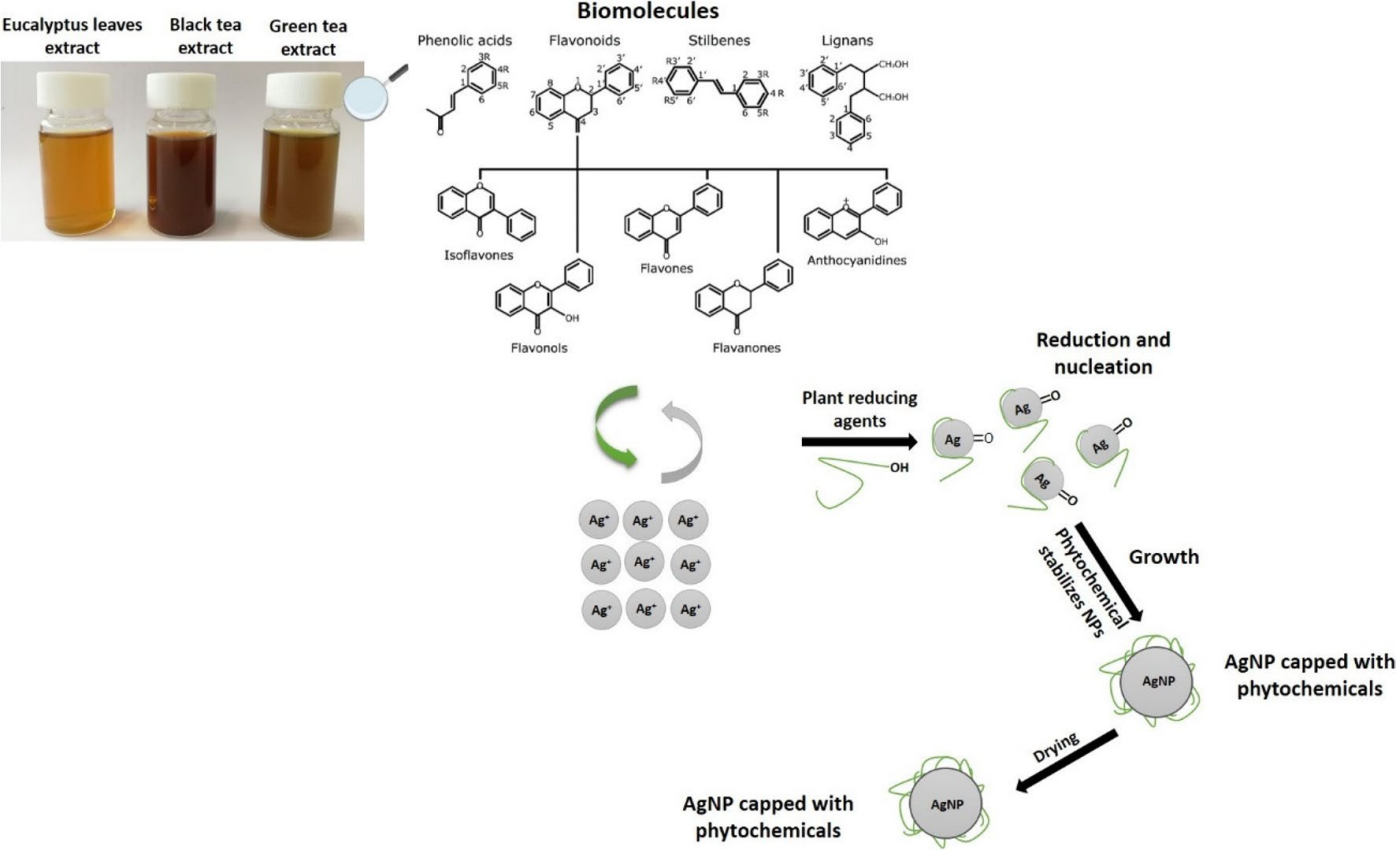
Schematic illustration of the mechanism of plant-mediated synthesis of silver nanoparticles (AgNP). Adapted from (Imran Din and Rani 2016)

Although the use of plant extract in biosynthesis has been extensively studied, the mechanism of bio-reduction of metal ions remains unknown (Mustapha et al. 2022). It has been proposed that the electrostatic trapping of silver ions on the surface of proteins in plant extract is the first step in silver bio-reduction (Marslin et al. 2018). The involvement of the secondary metabolites has also been proposed to imply the reduction of metal ions that lead to nanoparticle formation and support their subsequent stability (Mustapha et al. 2022). Figure 5 displays the probable mechanism of plant-mediated synthesis of AgNP from AgNO_3_ as suggested by Din and Rani (Imran Din and Rani 2016). It is proposed that the hydroxyl group of the various biomolecules present in the different extracts is primarily responsible for the reduction of silver ions (Ag^+^) into zero metallic species (Ag^o^). These Ag^o^ nuclei formed rapidly undergo the phenomenon of coalescence, culminating in the formation of AgNP.

### Antibacterial activity of aqueous extracts and AgNP

The antibacterial action of aqueous extracts from eucalyptus leaves, green tea and black tea and the respective biosynthesized AgNP was investigated against both Gram-negative (*P. putida*, *E. coli*, *Vibrio spp*.) and Gram-positive (*B. megaterium*, *S. aureus*, *S. equisimilis*) bacteria strains. The results of the inhibition zone (mm) are expressed in Table 2. The extracts were only effective against *Vibrio spp*. and the eucalyptus leaves extract showed the highest inhibition (11.5 ± 0.5 mm). In other studies, the application of *Eucalyptus globulus* extract showed an inhibition zone for *S. aureus* and *E. coli* (Balčiūnaitienė et al. 2022). However, the extracts were obtained with ethanol 70% (v/v), which may present different concentrations and profiles of bioactive compounds with antibacterial properties. In fact, ethanol is widely considered the ideal solvent for the extraction of phenolic compounds, although it is a flammable and costly solvent (Ferreira-Santos et al. 2020). So, water was used in this work as the safest, cheapest and most environmental friendly extraction solvent.

**Table 2:** Inhibition zones of the aqueous plant extracts and of silver nanoparticles (AgNP) against Gram-positive and Gram-negative bacterial strains

Bacterial strains		Inhibition zone (mm)					
		Aqueous extracts			AgNP		
		Eucalyptus leaves (EL)	Green tea (GT)	Black tea (BT)	EL-AgNP	GT-AgNP	BT-AgNP
Gram -	*E. coli*	n.d	n.d	n.d	10 ± 0.7^aA^	7.8 ± 0.4^bA^	10.3 ± 1.1^aA^
	*P. putida*	n.d	n.d	n.d	10.8 ± 0.4^aA^	7.8 ± 0.4^bA^	10.5 ± 0.5^aA^
	*Vibrio spp.*	11.5 ± 0.5^a^	10 ± 0^b^	10.5 ± 0.5^b^	14 ± 1^aB^	8.3 ± 0.8^bA^	12 ± 0^cB^
Gram +	*S. aureus*	n.d	n.d	n.d	13.5 ± 0.8^aB^	11.3 ± 0.8^bB^	12.8 ± 0.4^aB^
	*B. megaterium*	n.d	n.d	n.d	10.0 ± 0^aA^	7.3 ± 0.4^bA^	8.8 ± 0.8^cC^
	*S. equisimilis*	n.d	n.d	n.d	13.5 ± 0.5^aB^	7.8 ± 0.8^bA^	12.5 ± 0.5^aB^

n.d. = not detected; Values are expressed as mean (mm) ± SD; Different lowercase letters show significant differences (*p* < 0.05) between inhibition zone of aqueous extracts or AgNP for the same bacteria strain. Different capital letters show significant differences (*p* < 0.05) between bacterial strains for the same AgNP

The antibacterial action of the biosynthesized AgNP against the Gram-positive and Gram-negative bacteria can be appreciated in both cases. EL-AgNP, GT-AgNP and BT-AgNP were potentially effective in suppressing bacterial growth within a range of inhibition zones from 7.3 ± 0.40 to 14.0 ± 1 mm. EL-AgNP and BT-AgNP exhibited significantly (*p* < 0.05) higher antimicrobial activity than the GT-AgNP for all bacterial strains, but for *Vibrio spp*. and *B. megaterium*, for which EL-AgNP showed a higher inhibitory effect than BT-AgNP. The inhibition zones of EL-AgNP ranged between 10 ± 0 to 14 ± 1 mm. EL-AgNP showed a higher inhibition against *Vibrio spp*. (14 ± 1 mm), *S. aureus* (13.5 ± 0.8 mm) and *S. equisimillis* (13.5 ± 0.5 mm). In the case of *Vibrio spp.*, this higher inhibition has the contribution of the EL extract, since EL extract also shows antibacterial activity (11.5 ± 0.5 mm), while the remaining unique responsible for the effect is silver. Similar outcomes were obtained by (Liaqat et al. 2022)*,* who reported the antibacterial activity of AgNP biosynthesized by eucalyptus leaves against *E. coli* (8.0 ± 6 mm) and *S. aureus* (13.0 ± 0.3 mm). The inhibition zones of GT-AgNP ranged between 7.3 ± 0.4 to 11.3 ± 0.8 mm and the highest inhibition was shown against *S. aureus* (11.3 ± 0.8 mm). Previous studies reported antibacterial activity from green tea extract-mediated AgNP against *E. coli* and *S. aureus*, forming inhibition zones of 11 and 7 mm, respectively (Bergal et al. 2022). The inhibition zone of BT-AgNP ranged between 8.8 ± 0.8 to 12.8 ± 0.4 nm. BT-AgNP showed higher inhibition against *Vibrio spp*. (12 ± 0 mm), *S. aureus* (12.8 ± 0.4 mm) and *S. equisimillis* (12.5 ± 0.5 mm). The BT extract is also active against *Vibrio spp*. (10.5 ± 0 mm), enhancing the inhibitory effect obtained by BT-AgNP.

In general, the antibacterial action is higher against Gram-positive (*S. aureus* and *S. equisimillis*), likewise reported by other authors (Pirtarighat et al. 2019). As suggested by (Loo et al. 2018), Gram-negative bacteria may be less vulnerable due to the positive charges of AgNP trapped and inhibited by lipopolysaccharides, thus making them less susceptible.

It is suggested that AgNP demonstrated enhanced toxicity towards bacterial strains due to the small size and large intake of AgNP by bacteria (Ijaz et al. 2022). Although the detailed mechanism by which AgNP exhibits antibacterial action is not entirely known, various mechanisms of action have been documented in the bibliography. Due to their anchoring capabilities, AgNP have been demonstrated to induce structural changes in the bacterial membrane and eventual cell death as a result of their penetration into the cell wall (SINGH et al. 2016; Nguyen et al. 2020). Breakage of genetic material, inactivation of structural proteins and enzyme degradation by AgNP have been suggested as mechanisms of action too (Guzman et al. 2012). Another proposed mechanism is based on the release of silver cations from AgNP and on their interaction which results in severe changes in the bacterial membrane structure, increasing its permeability (Pirtarighat et al. 2019).

This antibacterial screening confirmed that green AgNP possess efficient antimicrobial potential against bacteria and could be used as an alternative disinfectants in wastewater remediation. The perfect disinfectant should be able to inactivate a wide range of microorganisms quickly, not corrosive, without producing any dangerous byproducts, using little energy and being easy to use and store. It should also be able to be safely disposed (Taher et al. 2022).

### Photocatalytic activity of EL-AgNP

Eucalyptus leaves, a biomass residues of the eucalyptus plantations, were considered a good source of bioactive compounds for the synthesis of AgNP. For this reason, the photocatalytic potential of biosynthesized EL-AgNP was assessed by evaluating the photodegradation of a model dye compound (indigo carmine (IC)), under direct sunlight. IC is an anionic (acidic)-type dye and it is one of the oldest dyes still widely used in many industries especially in textile, food, cosmetics, pharmaceuticals and medical diagnostics (Ammar et al. 2006; Ahirwar et al. 2016). It is a very toxic member of the indigoid dye class and direct contact causes eye and skin irritation, as well as permanent damage to the cornea and conjunctiva (Abdel Messih et al. 2017).

**Fig. 6:**
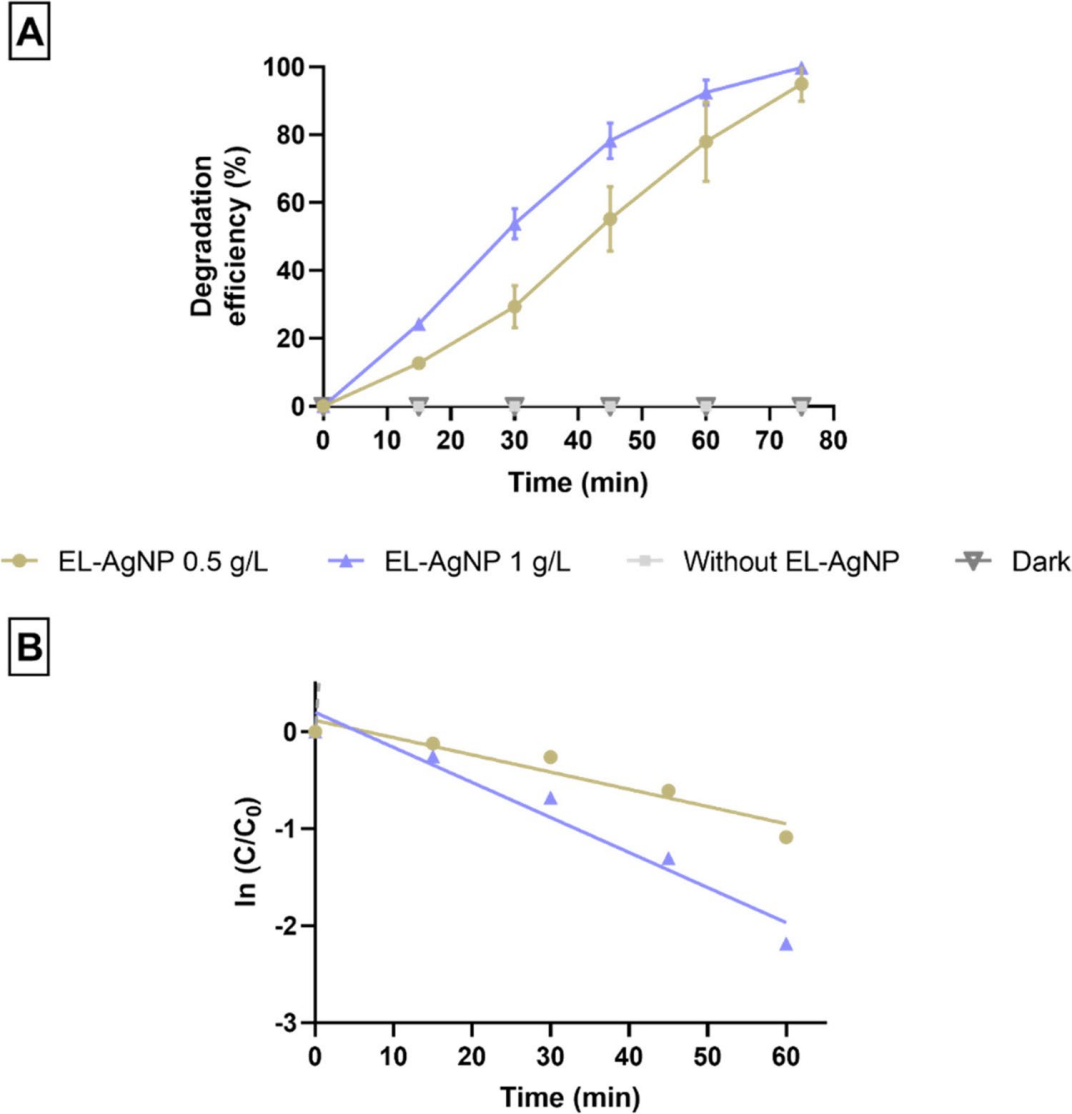
Degradation efficiency of indigo carmine (IC) dye by biosynthesized EL-AgNP (**A**) and pseudo-first-order reaction kinetics for IC decolorization by EL-AgNP 0.5 g/L (R^2^ = 0.9192) and EL-AgNP 1 g/L (R.^2^ = 0.9513) (**B**)

Figure 6A illustrates the photocatalytic degradation of IC dye under different dosages of EL-AgNP (0.5 and 1 g/L). Control experiments were performed to verify the effectiveness of EL-AgNP and the effects of direct sunlight on IC degradation. Degradation of IC was insignificant under direct sunlight and in the absence of AgNP. In addition, no significant changes were observed in the dye removal experiment conducted in the dark and in the presence of EL-AgNP. These findings indicate that the dye degradation should depend on both EL-AgNP and sunlight. The degradation percentages of the IC dye are nearly 95 and 100% under natural sunlight after 75 min with 0.5 and 1 g/L of EL-AgNP, respectively. The apparent kinetic parameters were estimated assuming that the degradation rate follows a pseudo-first-order kinetics represented by Eq. 4, (Fig. 6B). The catalytic reaction kinetics were evaluated through the creation of a graph of normalized concentration (C_t_/C_0_) *vs* time (Eq. 4).

4 $${\text{ln}}\frac{{C}_{t}}{{C}_{0}}=-k. t$$

C_0_ is the IC dye initial concentration, C_t_ is the concentration of IC dye at t (time) and k is the rate constant.

These kinetics assessments confirmed that the photocatalytic reaction follows a pseudo-first-order relationship, with an apparent rate constant (k) of 0.0178 and 0.03612 min^−1^ using 0.5 and 1 g/L of EL-AgNP, respectively. The dosage of 1 g/L led to the highest photocatalytic activity, indicating that the amount of catalyst influences the degradation rate as more electron-pairs are formed which increases the rate of reaction (Saeed et al. 2022).

Although it is difficult to compare the obtained results with those of the literature, as efficiency is a vital factor for the large scale of a photocatalyst, the photodegradation of IC with AgNP and other catalysts is presented (Table 3). Visible-light photocatalysis using sunlight as a light source in wastewater treatment has been regarded as an economic and an effective technology (Zheng et al. 2020). Very good results were obtained in the photodegradation of IC under sunlight exposure using AgNP on reduced graphene oxide (Ag-rGO) as catalyst (Jose et al. 2018), in the presence of sodium borohydride to initiate the photodegradation process. Also, Alsohaimi and co-workers (2020) studied the decolorization of IC using silver nanoparticles imprinted calcium oxide (Ag-CaO) under sunlight. The authors reported a degradation efficiency of 99.5%, with a noticeable adsorption (75%) of IC dye was observed. Faisal and colleagues (2020) tested the photodegradation of IC using green AgNP and obtained 98.2% of dye removal within 140 min under UV light. A higher degradation rate was found for the Ag-TiO_2_ composite, 0.044 min^−1^, (Abdel Messih et al. 2017), similar to our work (0.036 min^−1^). These results encourage further efforts to recognize the potential of EL-AgNP to photodegrade pollutants and to identify the respective mechanism of photodegradation.

**Table 3:** Comparative performance of silver nanoparticles (AgNP) and their composites for photodegradation of indigo carmine (IC) dye

Catalyst	Method of synthesis	Catalyst dosage (g/L)	IC concentration(mg/L)	Light source	Reaction time (min)	Degradation efficiency (%)	Degradation rate (min^−1^)	Ref
Ag-ZnO	Chemical co-precipitation	1.0	10	Visible	120	96	0.020	(Kumar et al. 2023)
Ag-ZnO	Sol–gel spin coating	n.r	6.6	Simulated sunlight	360	85	0.004	(Khiari et al. 2021)
Ag-CaO	Thermal	0.8	25	Sunlight	25	99.45	0.180	(Alsohaimi et al. 2020)
Ag-rGO	Hydrothermal	0.3	20	Sunlight	240	100	0.012	(Jose et al. 2018)
Ag-GO	Photochemical	1.0	10	Visible	420	54	0.003	(Martínez-Orozco et al. 2013)
Ag-TiO_2_	Sol–gel	1.0	28	UV	240	100	0.044	(Abdel Messih et al. 2017)
Ag-PbMoO_4_	Sonochemical	2.0	20	Simulated sunlight	120	n.a	0.026	(Gyawali et al. 2013)
Ag	Green using *Flammulina velutipes* extract	0.7	20	UV	140	98.2	n.r	(Faisal et al. 2020)
Ag	Green using *Eucalyptus globulus* leaves extract	1.0	10	Sunlight	75	99.8	0.036	This work

**Fig. 7:**
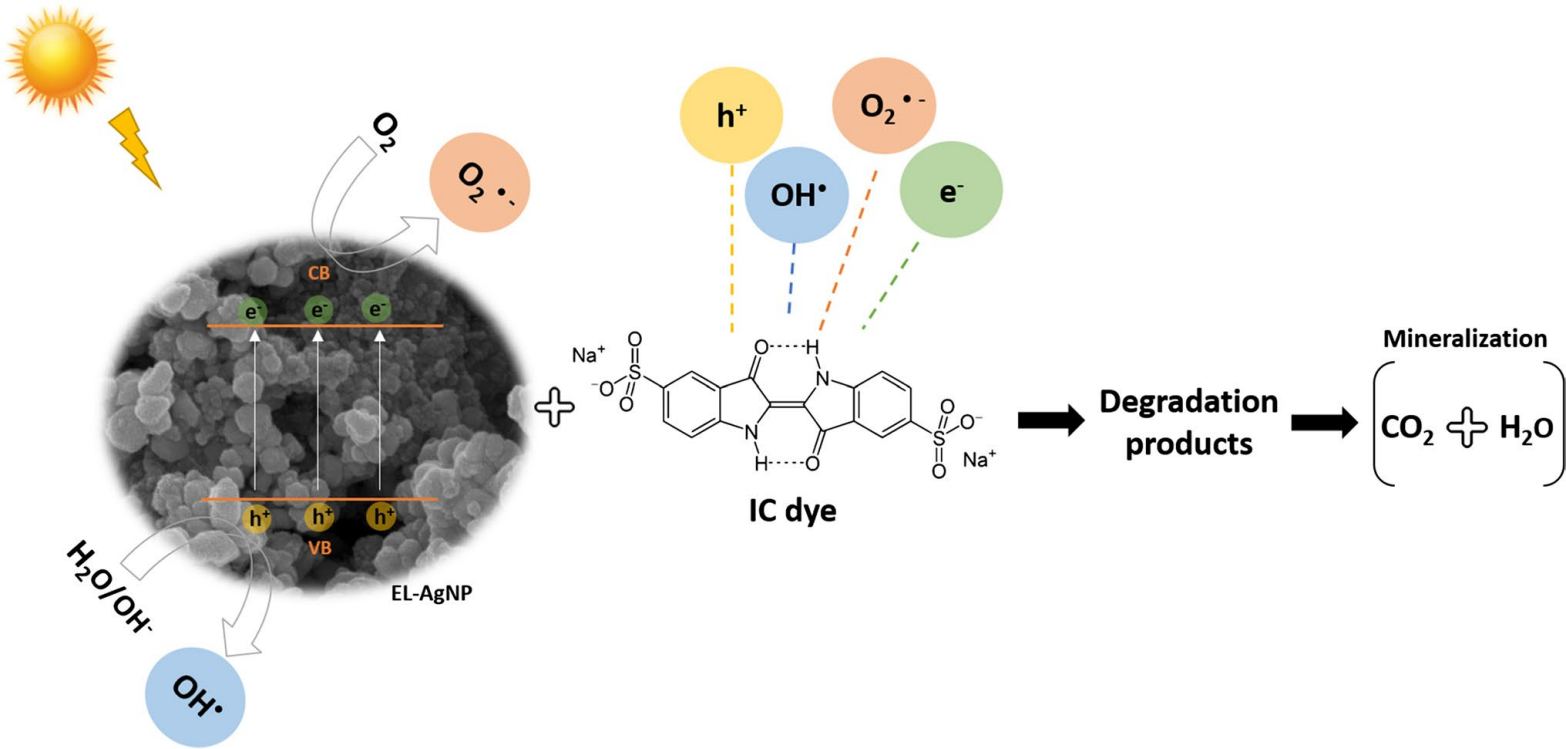
Schematic mechanism for photocatalytic degradation of IC dye by biosynthesized EL-AgNP

Figure 7 shows the schematic mechanism for photodegradation of IC dye by EL-AgNP under sunlight. The process is activated by the radiation of sunlight on EL-AgNP, which causes the excitation of electrons (e^−^) from the valence band (VB) to conduction band (CB) due to which generation of holes (h^+^) occurs in VB. These e^−^ and h^+^ will migrate to the surface of ELE-AgNP. The oxidation of OH^−^ in water by photo-generated h^+^, results in the generation of hydroxyl radicals (OH^•^). The e^−^ interacts with the dissolved oxygen (O_2_) and superoxide radicals (O_2_^•−^) are generated. These radicals react with IC dye to form degraded pollutants and ideally H_2_O and CO_2_ as final products. Some previous studies showed the photodegradation of IC dye into isatin sulfonic acid up to 2-amine-5-sulfo-benzoic acid formation via oxidation (Guaraldo et al. 2013; Hernández-Gordillo et al. 2016).

### Cost estimation of AgNP biofabrication

**Fig. 8:**
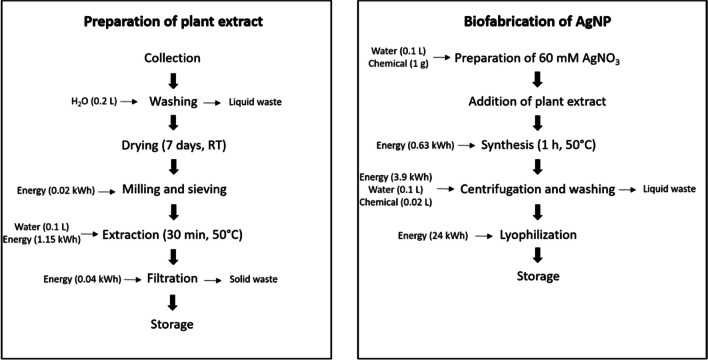
Flowchart for the preparation of 1 g of green AgNP

Successful implementation of materials usage for the elimination of pollutants and microorganisms from water depends largely on the cost of the material production. The cost assessment of the synthesis of green AgNP is a very important step to evaluate its cost-effectiveness and scalability of production. The total cost of the biosynthesis of AgNP covers various items including collection, washing, drying and milling of plant material; preparation and filtration of plant extract; and synthesis, centrifugation, washing and lyophilization of AgNP. Figure 8 shows the different steps performed to produce 1 g of AgNP as well as the input flows of energy, water and chemicals.

The total cost involved in this process was evaluated by determining the cost of energy, water, metallic salt and ethanol required for the biofabrication of AgNP. The cost of each step and the total preparation cost for 1 g of AgNP are given in Table 4.

**Table 4:** Estimation cost of preparation of 1 g of AgNP

	Plant Costs (€/g AgNP)	Eucalyptus leaves	Green tea	Black tea
Energy consumption^a^	Material collection	-	0.82	0.65
	Milling and Sieving	0.002	-	-
	Extraction	0.184	0.184	0.184
	Filtration	0.007	0.007	0.007
	Synthesis	0.1	0.1	0.1
	Centrifugation	0.624	0.624	0.624
	Lyophilization	3.84	3.84	3.84
Water consumption^b^	Plant washing, extraction process, AgNO_3_ solution and AgNP washing	0.0003	0.0003	0.0003
Chemicals	Silver nitrate^c^	2.78	2.78	2.78
	Ethanol^d^	0.4	0.4	0.4
	Total cost (€/g AgNP)	7.94	8.76	8.59

^a^Considering an energy cost of 0.16 €/kWh; ^b^Considering a cost of 0.70 €/m^3^ of deionized water; ^c^Considering a cost of 69.60 €/25 g and ^d^Considering a cost of 16.02 €/2.5 L

The cost associated with the collection of eucalyptus leaves was not considered once it is locally and abundantly available and was acquired as a biowaste. Green and black tea were purchased as commercial products (Tetley®) and were used as received. The cost of material collection, energy, water and chemicals was defined considering average values practiced in Portugal.

The total cost of the synthesis of green AgNP in the laboratory ranges 7.94–8.76 €/g. The main items with a remarkable cost are the energy required in the lyophilization step and the silver nitrate. The commercial AgNP are currently priced between 18.48 €/g (Sigma-Aldrich, #576,832) and 28.72 €/g (ThermoFischer Scientific, #045509.14), which illustrates the need to develop cheaper and scalable alternatives. This cost analysis reveals that the proposed green synthesis is comparatively very competitive.

## Conclusions

The use of plant extracts for the biosynthesis of AgNP is an alternative green method of preparation due to its quick, ecological, non-pathogenic and inexpensive procedure. From all plant by-products tested, the EL, GT and BT extracts obtained at 50 °C for 30 min, were the ones with lower associated energy costs and were selected as promising candidates to biosynthesize AgNP. Here the importance of the previous optimization of the extraction process was emphasized, guaranteeing the extraction's efficiency and reducing energetic costs, making the process more sustainable. The resulting biosynthesized AgNP have antimicrobial activity against Gram-negative and Gram-positive bacteria, with EL-AgNP being the nanostructure with the greatest antimicrobial action. The photocatalytic capacity of EL-AgNP was proved by the total decolorization of the IC dye after 75 min of reaction under sunlight. The cost analysis demonstrated the potential of this green approach to enable the large-scale deployment of AgNP in a wide range of environmental applications. These results encourage the application of bio-resources in the synthesis of MNP and the study of their potential as multifunction agents for wastewater treatment.

### Supplementary Information

Below is the link to the electronic supplementary material.

Supplementary file1 (DOCX 20 KB)
