# Supplementary material for: Valorization of plant by-products in the biosynthesis of silver nanoparticles with antimicrobial and catalytic properties
Source: Environ Sci Pollut Res Int. 2024 Jan 26;31(9):14191–207. doi: 10.1007/s11356-024-32180-w (PMC10881659; doi:10.1007/s11356-024-32180-w)
Supplement: Supplementary file 1 — Supplementary file1 (DOCX 20 KB) [file 11356_2024_32180_MOESM1_ESM.docx]

**Valorization of plant by-products in the biosynthesis of silver nanoparticles with antimicrobial and catalytic properties**

Verónica Rocha^1*^, Pedro Ferreira-Santos^2^, Cristina Almeida Aguiar^3^, Isabel C. Neves^1,4^ and Teresa Tavares^1,5^

^1^ CEB - Centre of Biological Engineering, University of Minho, Campus de Gualtar, 4710-057 Braga, Portugal;

^2^ Department of Chemical Engineering, Faculty of Science, University of Vigo, As Lagoas, 32004 Ourense, Spain;

^3^ CBMA—Centre of Molecular and Environmental Biology, University of Minho, 4710-057 Braga, Portugal;

^4^ CQ-UM – Centre of Chemistry, University of Minho, Campus de Gualtar, 4710-057 Braga, Portugal;

^5^ LABBELS –Associate Laboratory, 4710-057 Braga/Guimarães, Portugal.

*Corresponding author: [vrocha@ceb.uminho.pt](mailto:vrocha@ceb.uminho.pt) (Verónica Rocha)

**Materials and Methodologies**

**Materials**

Silver nitrate (AgNO_3_) was obtained from PanReac AppliChem (Castellar del Vallès, Barcelona). Indigo carmine dye (5,5′-indigodisulfonic acid sodium salt, C_16_H_8_N_2_Na_2_O_8_S_2_) was acquired from Sigma-Aldrich (Lisboa, Portugal). The nutrient broth and agar for the antimicrobial assays were bought from Oxoid (Madrid, Spain). All other analytical-grade chemicals were acquired from Sigma-Aldrich. Ultrapure water was used in all experiments and solutions.

**Preparation of plant extract**

Eucalyptus bark and eucalyptus leaves (*Eucalyptus globulus*), pine needles (*Pinus pinaster*) and orange leaves (*Citrus sinensis*) were collected in Marco de Canaveses, Portugal. Cedar wood (*Cedrus atlantica*) was collected in Fez, Morocco. Green tea and black tea (*Camellia sinensis*) were purchased as commercial products (Tetley®) and were used as received. The collected plants were washed using distilled water to eliminate impurities adhered to leaves and dried in shade for 7 days, then they were chopped and ground to a fine powder (< 1 mm) in a mechanical grinder. Dried plant powder (2 g) was subjected to a conventional solid-liquid extraction using a cylindrical reactor into a water bath with continuous shaking (200 rpm) and extracted with 20 mL of water. Extractions were done at different temperatures (50 and 80 °C) and during different times (30 and 60 min). The obtained extracts were clarified through Whatman filter paper No. 1 and kept at 4 °C until use. All the experiments were done at least in triplicate.

**Characterization of AgNP**

A UV-Vis spectrophotometer (model V-630, JASCO) was used to evaluate the optical properties of biosynthesized AgNP at wavelengths between 350 and 700 nm. An ultra-high resolution field-emission scanning electron microscope (SEM, FEI Nova 200) with an integrated microanalysis X-ray system (EDX - energy dispersive X-ray spectroscopy, Pegasus X4M) was used to analyze the powder AgNP. The role of aqueous extracts and their interaction with AgNP were investigated by Fourier Transform Infrared Spectroscopy with a diamond-composite attenuated total reflectance cell (FTIR-ATR, ALPHA II-Bruker spectrometer). The zeta potential of AgNP was accessed by dynamic light scattering (DLS, Malvern Panalytical) at 25 °C, 150 V.

**Statistical Analysis**

All experiments were carried out on three independent tests and results were expressed as mean ± standard deviation (SD). The significance of the data was statistically determined with One-way ANOVA followed by Tukey´s multiple comparisons test using GraphPad Prism® software (version 8.0). *p* values < 0.05 were considered to be statistically significant.
